# Supplementary material for: hiPSC‐Derived Astrocytes From Individuals With Schizophrenia Induce a Dystrophic Phenotype in Microglial‐Like Cells
Source: Glia. 2025 Sep 19;74(1):e70085. doi: 10.1002/glia.70085 (PMC12666992; doi:10.1002/glia.70085)
Supplement: Supplementary file 1 — Figure S1: Supporting Information Figures. [file GLIA-74-0-s002.pdf]

SUPPLEMENTARY FIGURES

FIGURE S1

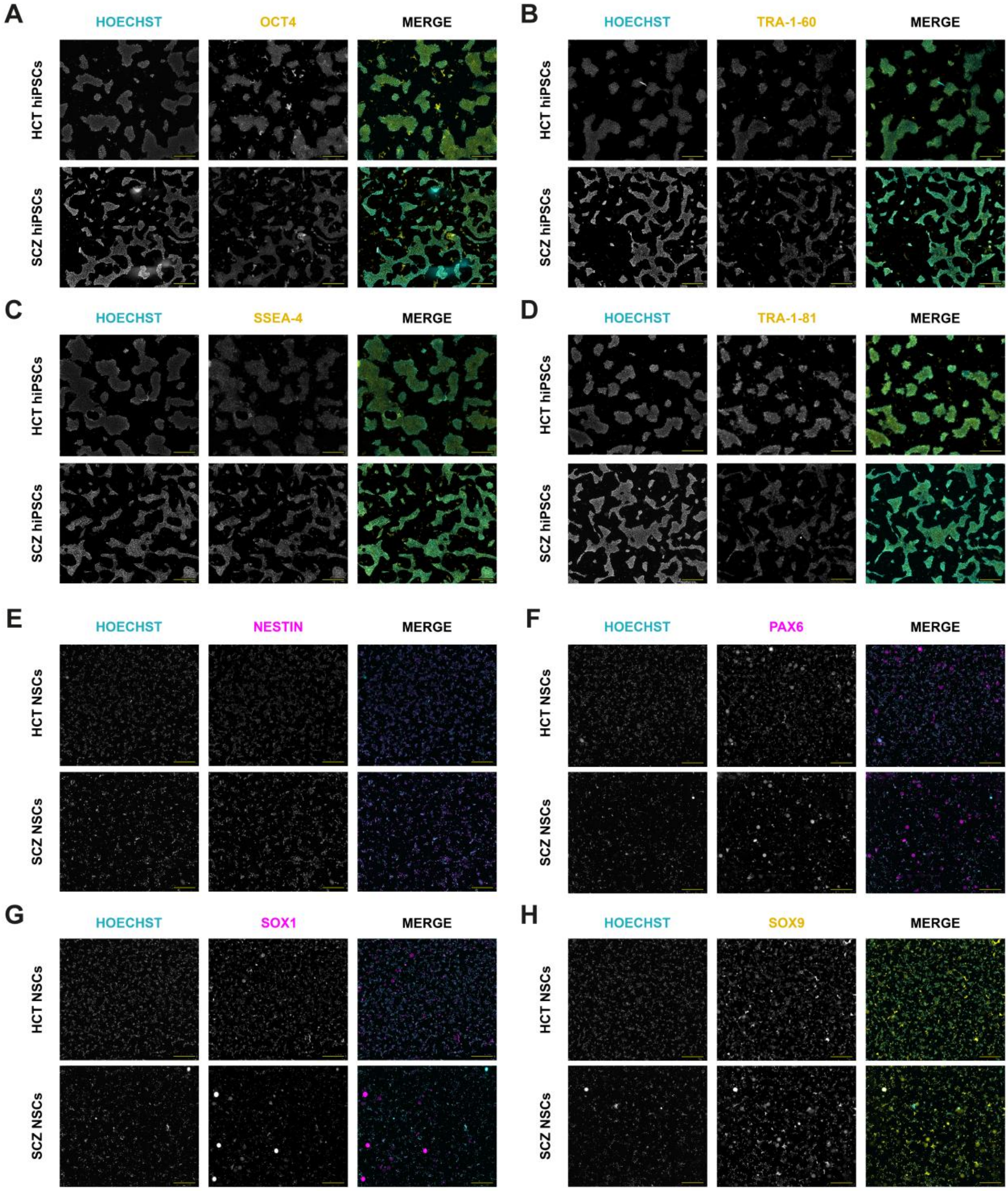

FIGURE S1: hiPSCs and NSCs characterizations. hiPSCs and hiPSC-derived NSCs sourced from HCT and SCZ individuals were characterized by immunostaining. **(A-D)** hiPSCs display positive staining for the pluripotency stem cell markers OCT4 (yellow; **A**), TRA-1-60 (yellow; **B**), SSEA-4 (yellow; **C**) and TRA-1-81 (yellow; **D**). **(E-H)** NSCs stains for the neural stem cell markers Nestin (magenta; **E**), PAX6 (magenta; **F**), SOX1 (magenta; **G**) and SOX9 (yellow; **H**). Nuclei were counterstained with Hoechst (cyan). *Scale bar = 700 µm.*

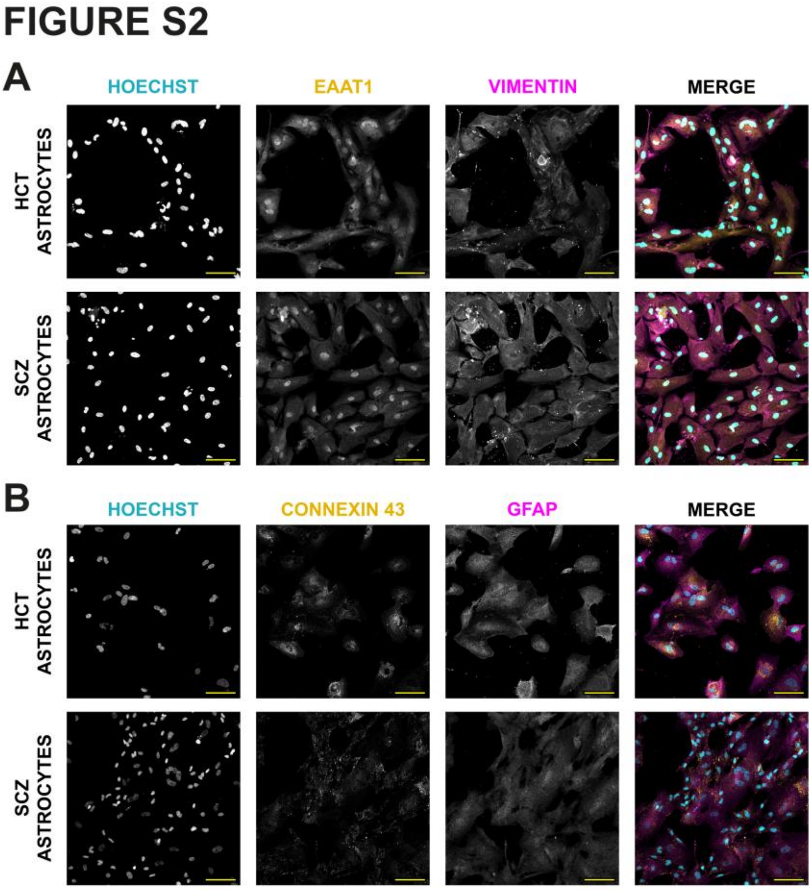

FIGURE S2: hiPSC-derived astrocytes characterization. (A-B) HCT and SCZ hiPSC-derived astrocytes were characterized by immunostaining. Astrocytes display positive staining for EAAT1 (yellow, **A**), Vimentin (magenta, **A**), Connexin 43 (yellow, **B**) and GFAP (magenta, **B**). Nuclei were counterstained with Hoechst (cyan). *Scale bar = 100 µm.*

FIGURE S3

A

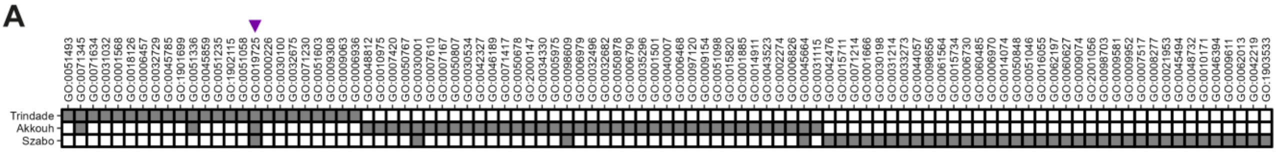

B

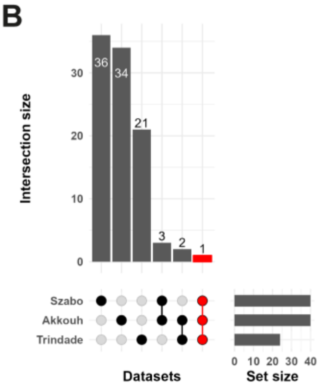

C

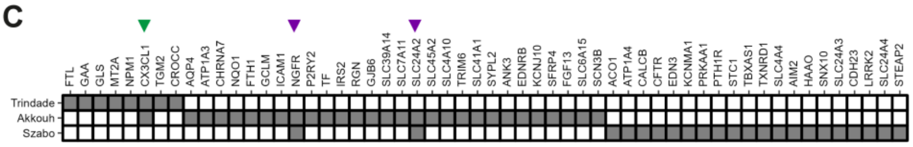

D

Gene Symbol	Akkouh (Log_2 FC)	Szabo (Log_2 FC)	Trindade (Log_2 FC)	GO Molecular Function
CX3CL1	↑↑ (2.558)	-	↑ (0.580)	Chemokine activity
NGFR	↑↑ (2.143)	↑↑ (3.341)	-	Nerve Growth Factor (NGF) binding
SLC24A2	↓↓ (-2.235)	↑↑ (2.563)	-	Calcium, Potassium:Sodium antiporter activity

E

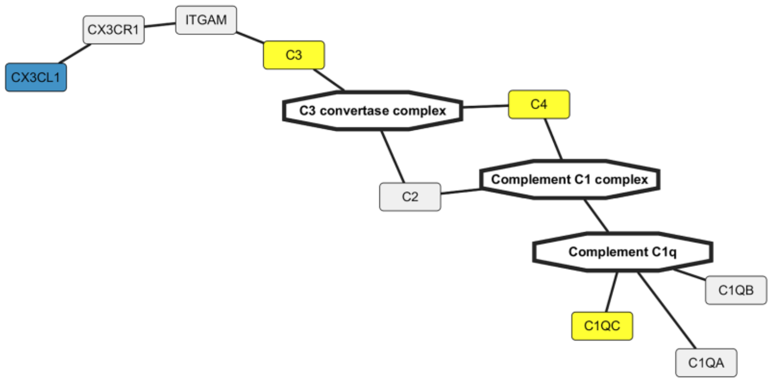

FIGURE S3: Candidate gene screening suggested a potential role for astrocyte-produced CX3CL1 in schizophrenia. (A) GO Biological Processes identified from Akkout *et al*, Szabo *et al* and Trindade *et al* datasets using *Metascape*. Filled and open squares indicate the enrichment or not of a given GO term in each dataset, respectively. Purple arrowhead points to GO:0019725 (cellular homeostasis), the only GO term to be enriched in the three studies. **(B)** Upset plot showing the number of overlapping enriched GO terms in all datasets. **(C)** Candidate genes extracted from GO:0019725 in each dataset. Purple arrowheads point to NGFR and SLC24A2 and green arrowhead points to CX3CL1, which has been chosen for subsequent investigation in the present study. **(D)** Table summarizing CX3CL1, NGFR, and SLC24A2 expression change in each dataset and their respective GO Molecular Function. **(E)** Synaptic pruning proteins network, highlighting CX3CL1 (blue) and classical complement components (yellow): C1q (subunit C), C3 and C4. Macromolecular complexes are drawn as hexagons and individual proteins as round rectangles.

FIGURE S4

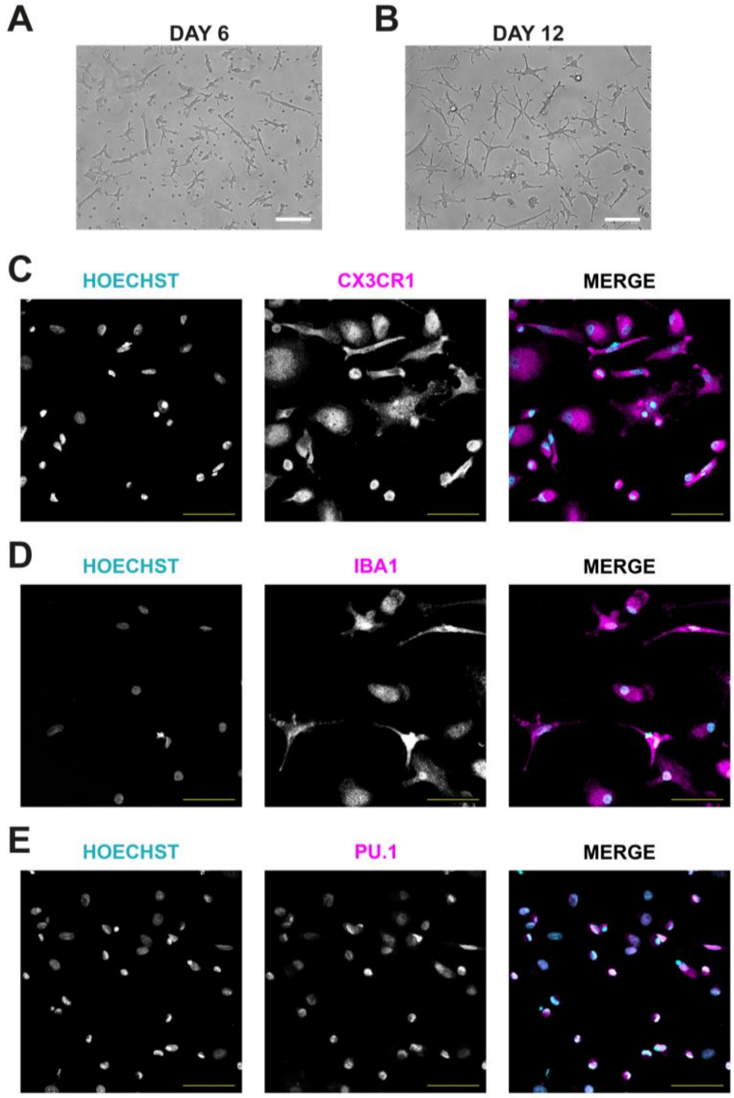

FIGURE S4: Induced microglial-like cells (iMGs) characterization. (A-B) Bright-field image of iMGs in their 6th **(A)** and 12th day of differentiation **(B)**, displaying a progressive ramified morphology. *Scale bar = 100 µm.* **(C-E)** 12-day differentiated iMGs were characterized by immunostaining. iMGs displayed positive staining for CX3CR1 (magenta, **C**), IBA1 (magenta, **D**) and PU.1 (magenta, **E**). Nuclei are counterstained with Hoechst (cyan). *Scale bar = 50 µm.*

FIGURE S5

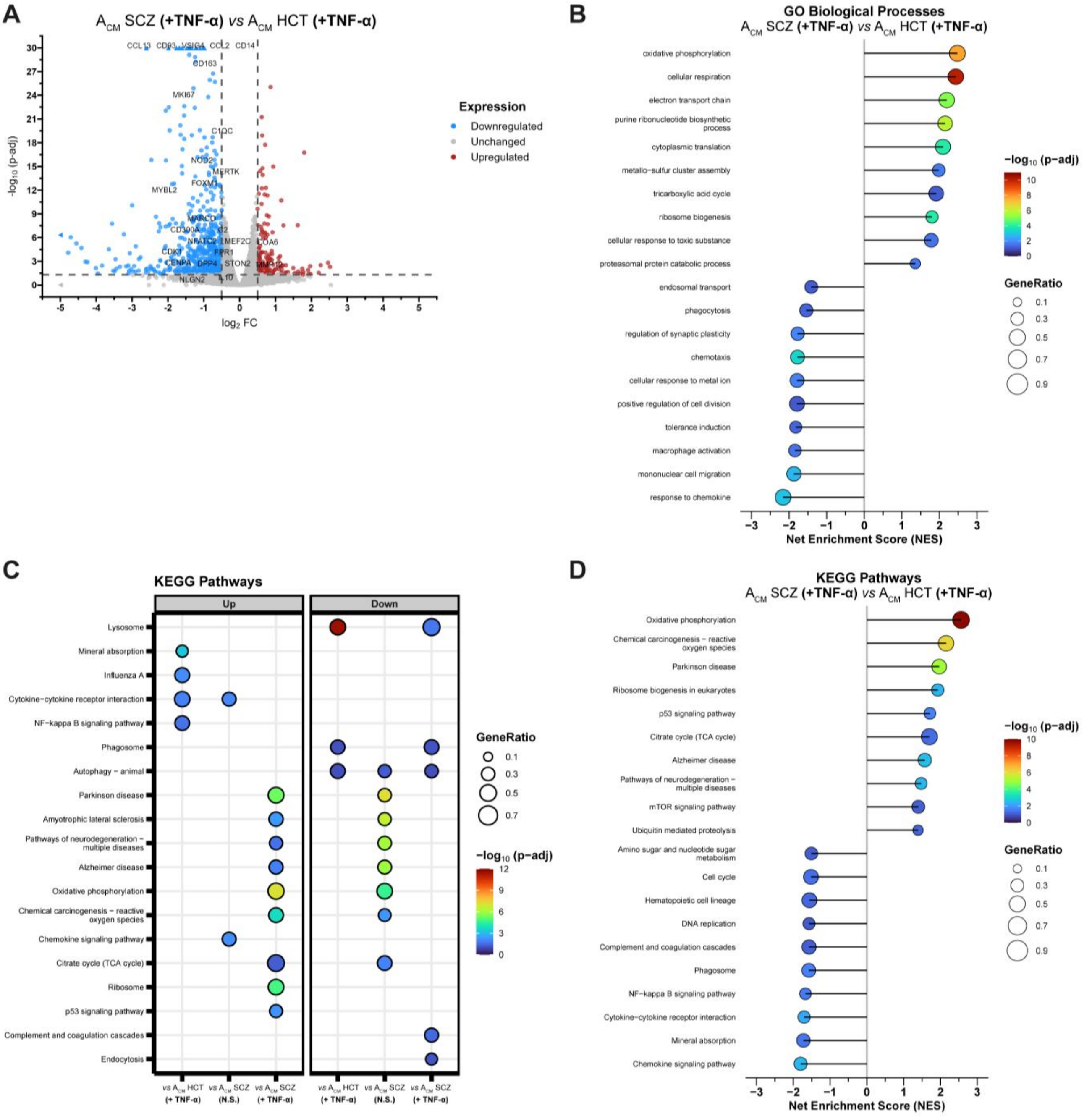

FIGURE S5: TNF-α-stimulated SCZ astrocytes induced the upregulation of pathways related to neurodegenerative diseases in iMGs. (A) Volcano plot depicting DEGs in iMGs cultured with ACM SCZ (+TNF-α) vs ACM HCT (+TNF-α). Upregulated and downregulated DEGs are colored in red and blue, respectively. Circles and triangles indicate genes within and out of the plot axes range, respectively**. (B)** Gene-set enrichment analysis (GSEA) plot results presenting the most relevant GO Biological Processes positively or negatively enriched in iMGs exposed to ACM SCZ (+TNF-α) vs ACM HCT (+TNF-α). **(C)** GSEA plot results showing the most relevant KEGG Pathways associated with each experimental condition. *vs means that the indicated analysis is expressed relative to iMGs + A_{CM} HCT (N.S.).* **(D)** GSEA plot results presenting the most relevant KEGG Pathways positively or negatively enriched in iMGs exposed to ACM SCZ (+TNF-α) vs ACM HCT (+TNF-α). *NES: Net Enrichment Score*.

FIGURE S6

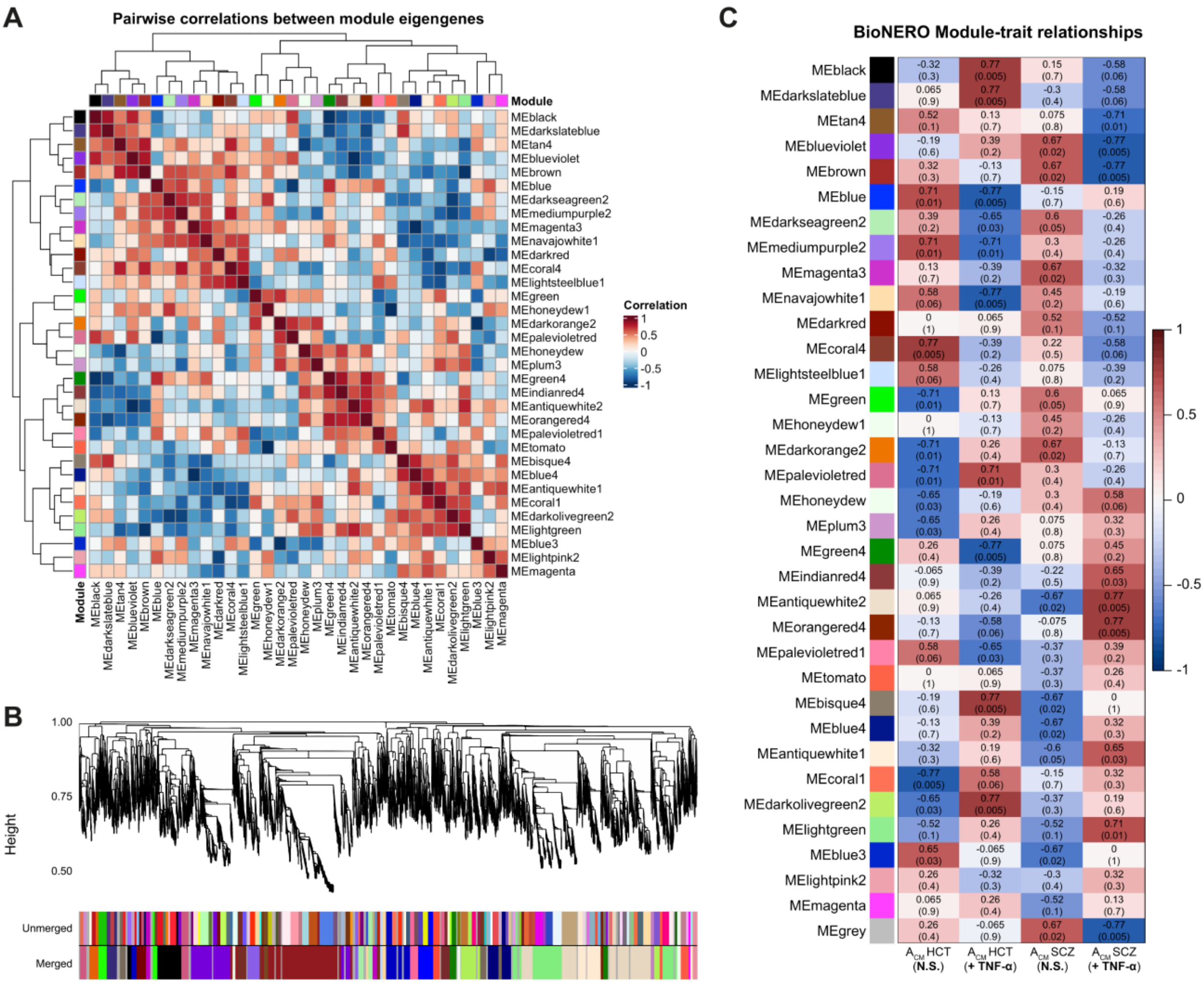

FIGURE S6: WGCNA revealed modules enriched for GO terms involved in endocytosis, phagocytosis, chemotaxis and cell migration processes. (A) Heatmap showing the pairwise correlation between all 35 module eigengenes. **(B)** Dendrogram depicting hierarchical clustering among identified co-expression modules. *Modules with Spearman correlation greater than 0.8 were merged.* **(C)** Module-trait relationships heatmap displaying the correlation between WGCNA co-expression module eigengenes and each experimental condition (i.e., iMGs + indicated ACM). *Numbers in parentheses indicate adjusted p-values; above them are shown correlation values.*

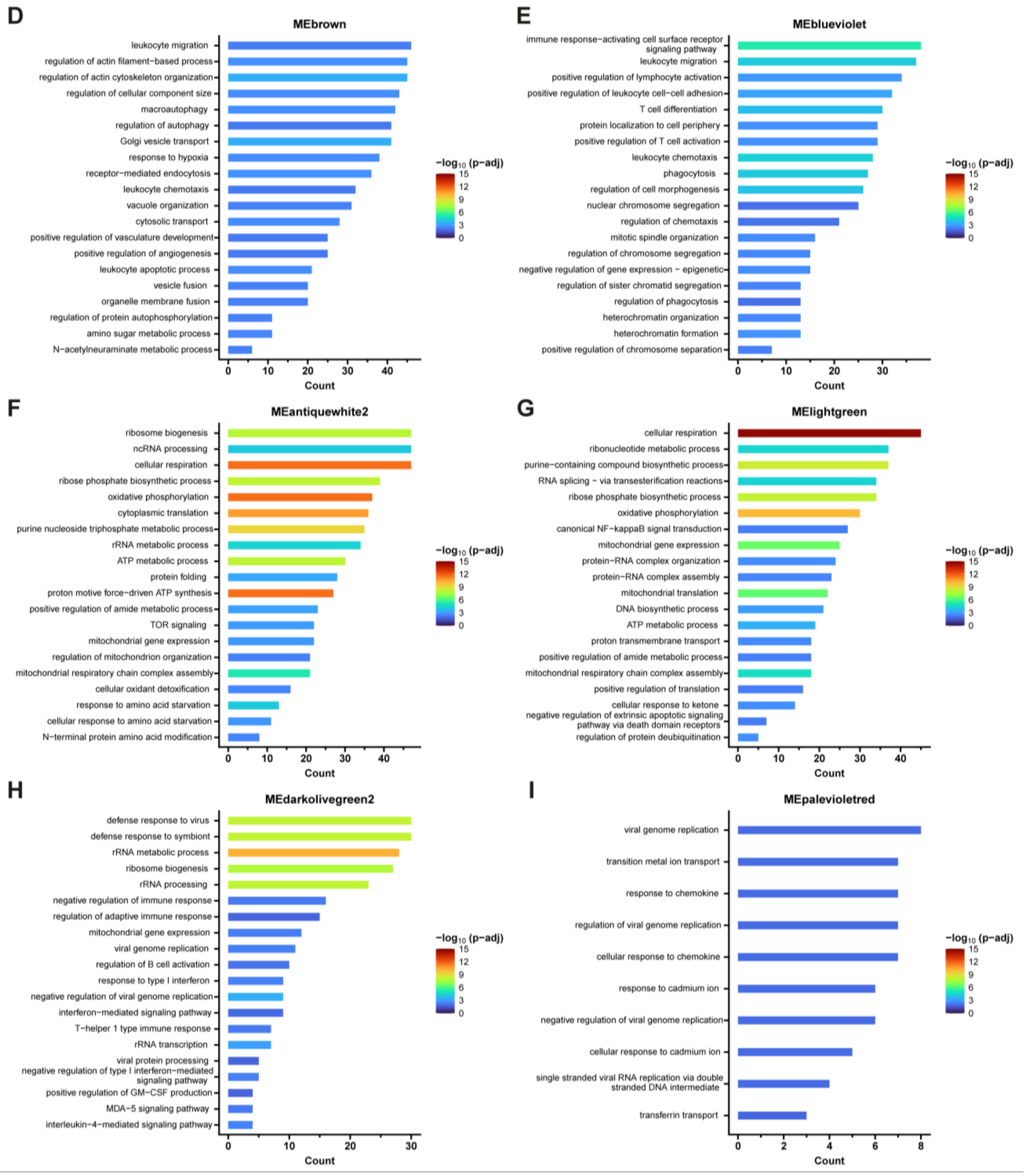

FIGURE S6 (continuation): (D-H) Top 20 GO Biological Processes enriched in the *brown* **(D)**, *blueviolet* **(E)**, *antiquewhite2* **(F)**, *lightgreen* **(G)** and *darkolivegreen2* **(H)** WGCNA modules. **(I)** Top 10 GO Biological Processes enriched in the *palevioletred* module.

FIGURE S7

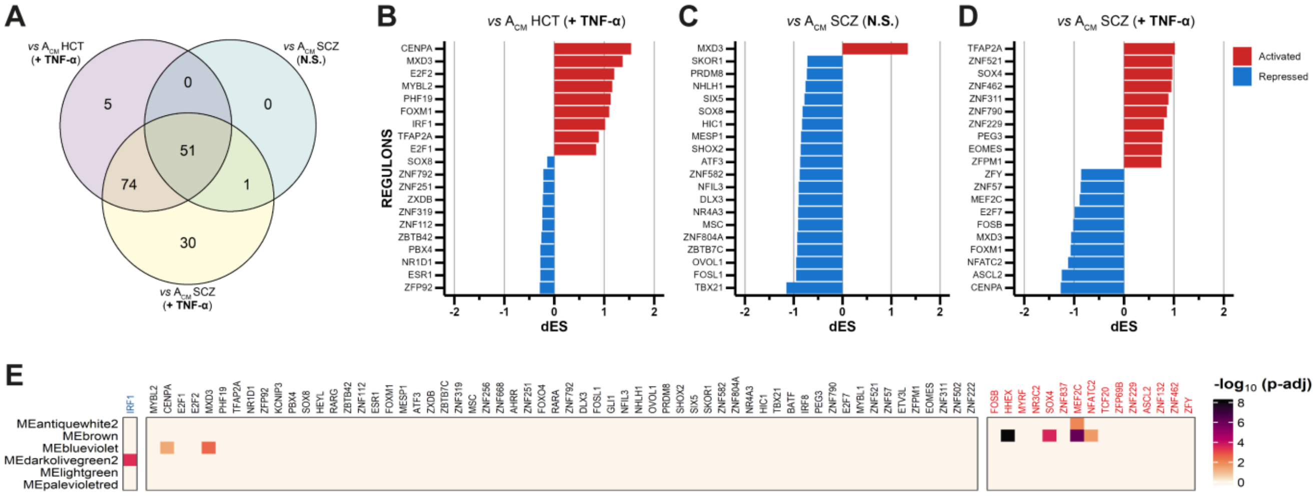

FIGURE S7: Master Regulator Analysis identified putative transcription factors regulating the transcriptional response in iMGs incubated with A_{CM}. (A) Venn diagram showing shared master regulators between iMGs exposed to A_{CM} HCT (+TNF-α), A_{CM} SCZ (N.S.), or A_{CM} SCZ (+TNF-α). **(B-D)** Top and bottom 10 regulons ranked based on their activity status in iMGs + A_{CM} HCT (+TNF-α) **(B)**, iMGs + A_{CM} SCZ (N.S.) **(C),** and iMGs + A_{CM} SCZ (+TNF-α) **(D)**. *dES: differential enrichment score. vs points that the indicated analysis is expressed relative to iMGs +* A_{CM} *HCT (N.S.).* **(E)** *In silico* master regulator analysis depicting putative transcription factors (*columns*), whose predicted target genes showed significant overlap with the indicated WGCNA modules gene sets (*rows*) shown in **Figure S6**. *Column names in blue or red indicate master regulators exclusively found in either iMGs +* A_{CM} *HCT (+TNF-α) or iMGs +* A_{CM} *SCZ (+TNF-α), respectively, while regulons shared by at least two experimental conditions are shown in black.*

FIGURE S8

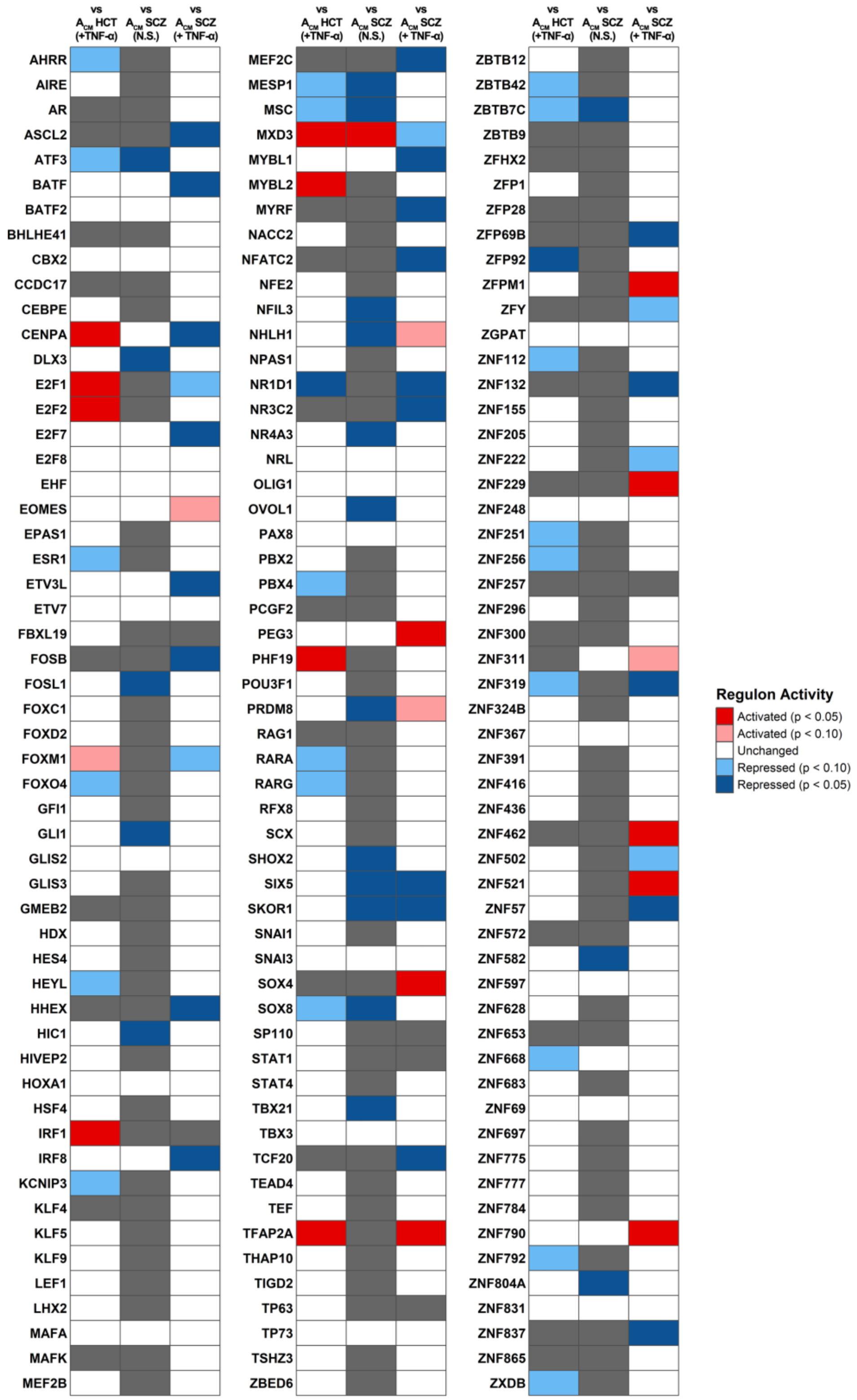

FIGURE S8: Regulon activity of master regulators. Regulon activity of master regulators is shown for each experimental group (iMGs incubated with A_{CM} HCT (+TNF-α), A_{CM} SCZ (N.S.) or A_{CM} SCZ (+TNF-α)). *Light blue: repressed ($p < 0.1$); dark blue: repressed ($p < 0.05$); pale red: activated ($p < 0.1$); red: activated ($p < 0.05$); white: unchanged ($p \geq 0.1$); gray: regulon not significantly associated with that experimental group.* *<u>vs</u> points that the indicated analysis is expressed relative to iMGs + A_{CM} HCT (N.S.).*

FIGURE S9

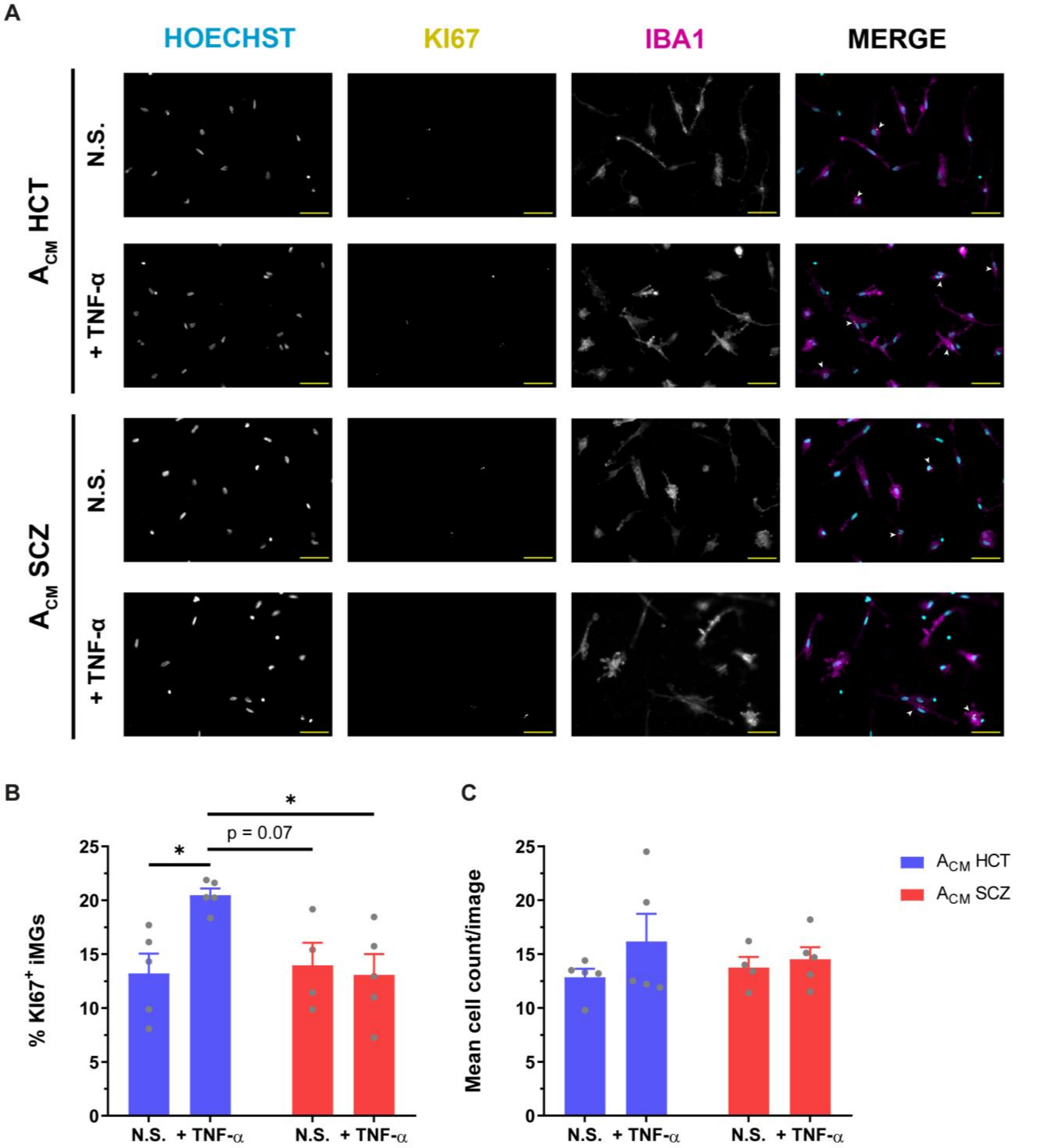

FIGURE S9: TNF-α-stimulated HCT astrocytes increase the amount of KI67^{+} microglial-like cells. (A) iMGs were incubated for 24 h with all four A_{CM} conditions and subsequently fixed and stained for KI67 (yellow) and IBA1 (magenta). Nuclei were counterstained with Hoechst (cyan). White arrowheads point to iMGs containing KI67^{+} puncta in merged images only. *Scale bar = 50 µm.* **(B)** Quantification of iMGs containing KI67^{+} puncta relative to panels shown in **(A)**. *n = 4-5 replicates (two independent experiments). Data were analyzed by One-way ANOVA, followed by Holm-Sidak's multiple comparison test. Bars represent Mean ± SEM.* **(C)** Quantification of cell count from panels shown in **(A)** and displayed as mean cell count/image per replicate. *n = 4-5 replicates (two independent experiments). Data were analyzed by One-way ANOVA, followed by Holm-Sidak's multiple comparison test. Bars represent Mean ± SEM.* * $p < 0.05$.

FIGURE S10

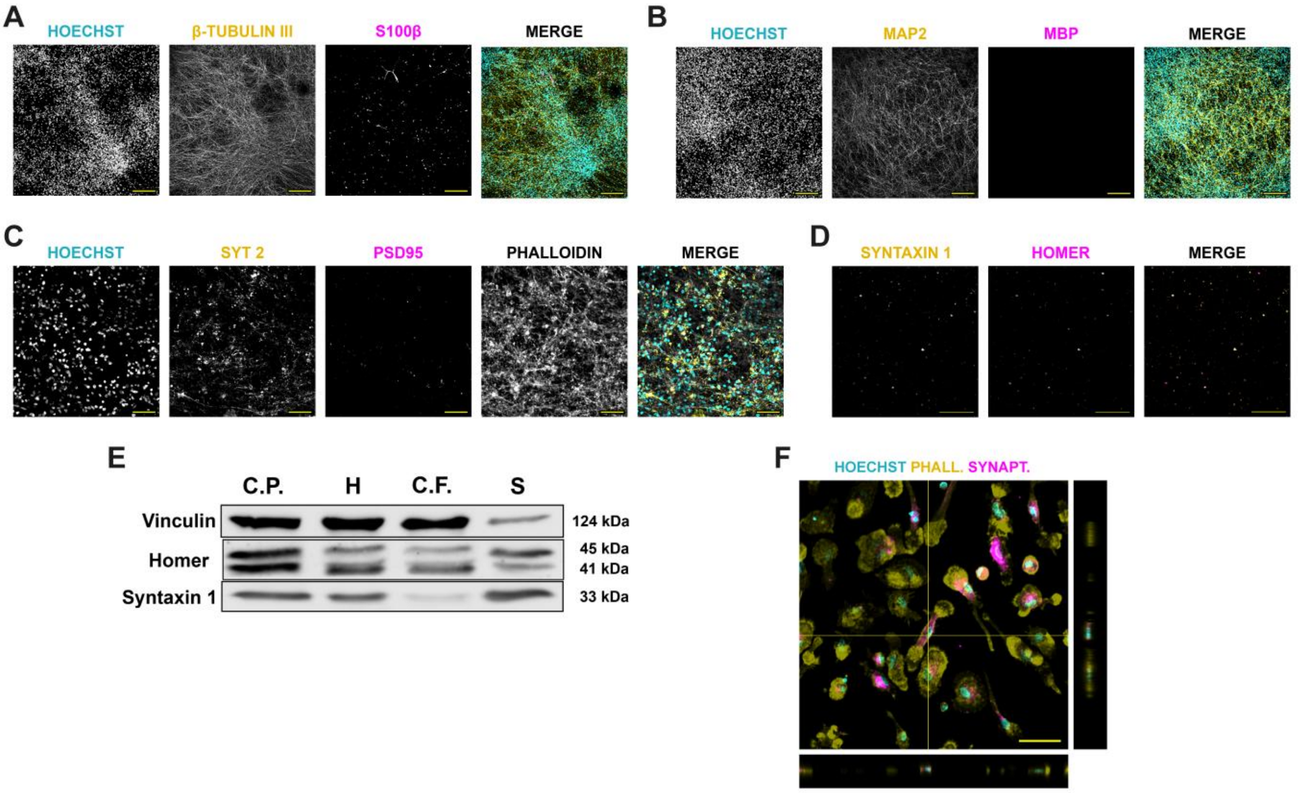

FIGURE S10: Synaptoneurosomes isolation from hiPSC-derived neurons and their engulfment by iMGs. (A-B) 60 days hiPSC-derived neuronal cultures stain for mature neuronal markers (β-tubulin III in **A**; MAP2 in **B**; yellow) and display few astrocytes (S100β in **A**; magenta) and no oligodendrocytes (MBP in **B**; magenta). Nuclei are counterstained with Hoechst (cyan). *Scale bar = 200 µm.* **(C)** Mature hiPSC-derived neurons show positive staining for presynaptic (SYT 2: Synaptotagmin 2; yellow) and postsynaptic (PSD95; magenta) markers. Cells were stained with Phalloidin (grey), and nuclei were counterstained with Hoechst (cyan). *Scale bar = 50 µm.* **(D)** Isolated synaptoneurosomes stained for the presynaptic marker Syntaxin 1 (yellow) and the postsynaptic marker Homer (magenta). *Scale bar = 50 µm.* **(E)** Western blot for Vinculin, Homer, and Syntaxin 1 of each fraction collected during synaptoneurosomes isolation procedure. *C.P.: cell debris pellet; H: homogenate; C.F.: cytosolic fraction; S: synaptoneurosomes.* **(F)** Representative image showing iMGs engulfing CM-DiI-labelled synaptoneurosomes (magenta), as shown in this orthogonal projection of a z-stack confocal microscopy image. iMGs are stained for Phalloidin (yellow) and nuclei are counterstained with Hoechst (cyan). *Scale bar = 50 µm.*

FIGURE S11

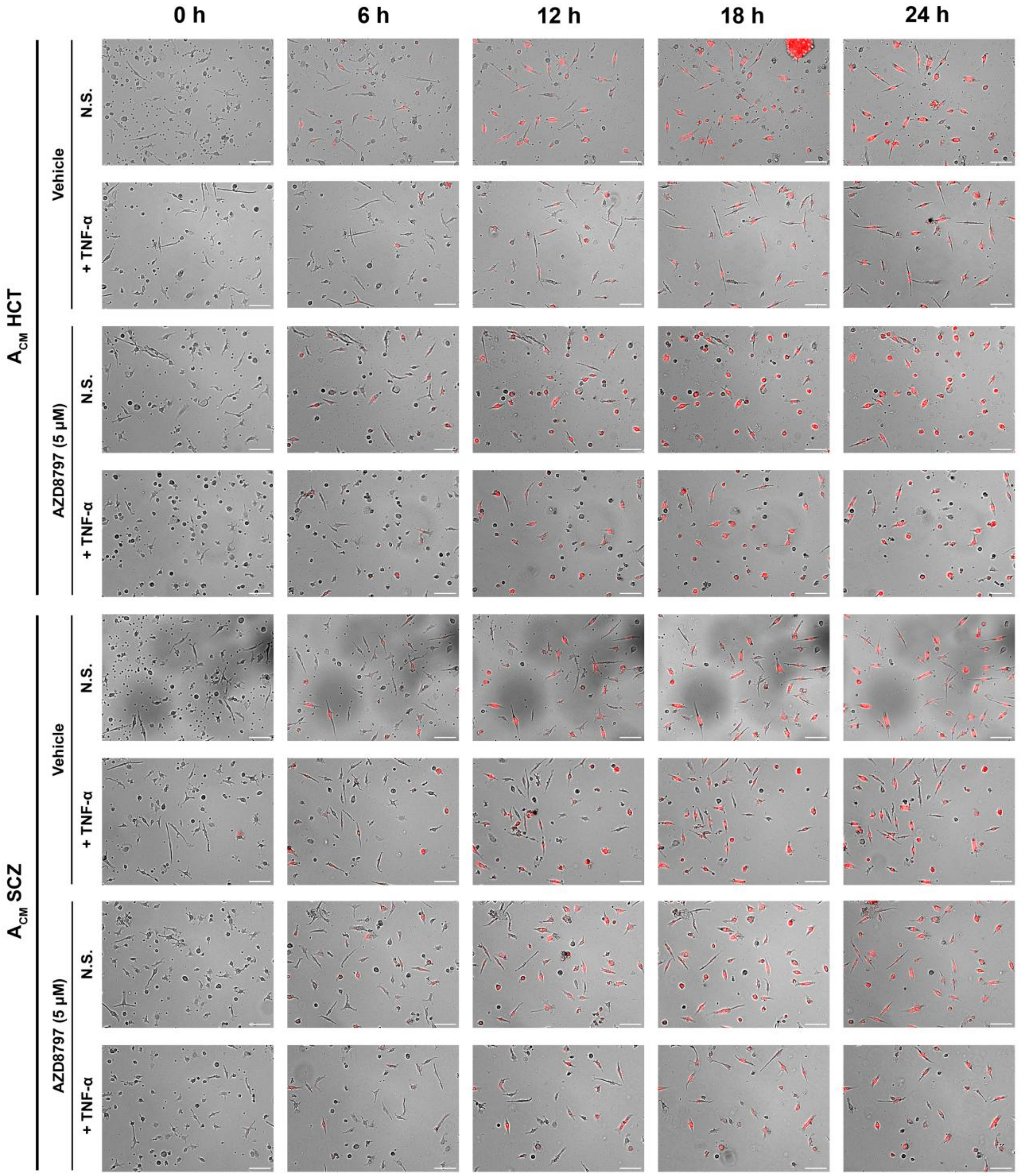

FIGURE S11: iMGs engulfing fluorescently labelled synaptoneurosomes after pre-treatment with AZD8797 and following exposure to A$_{CM}$. Full panel showing iMGs (bright field) phagocytosing CM-DiI-labelled synaptoneurosomes (red) upon incubation with A$_{CM}$ and pre-treatment with the CX3CR1 antagonist AZD8797 (5 µM). Panel depicting 0 h, 6 h, 12 h, 18 h, and 24 h time-points. *Scale bar = 100 µm.*

FIGURE S12

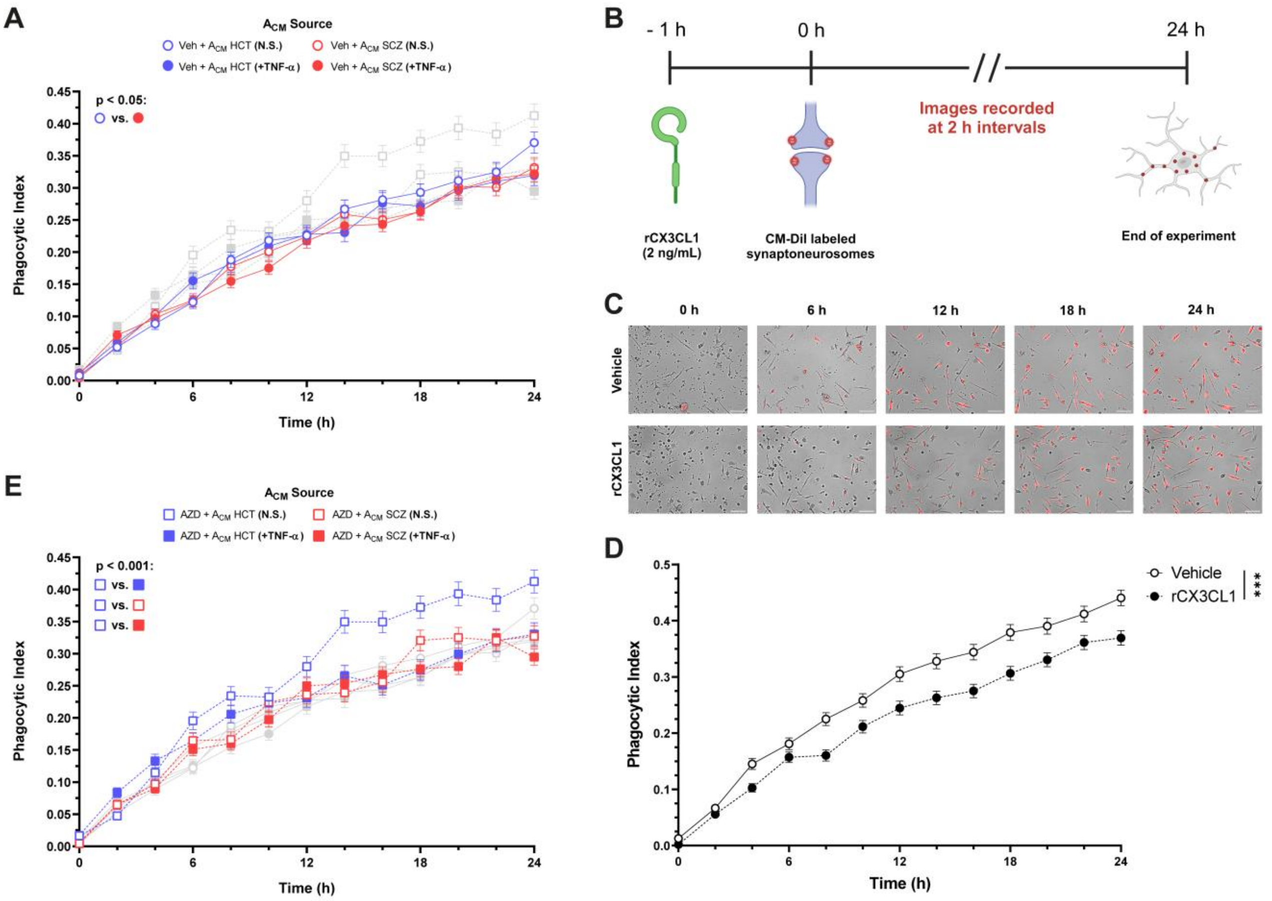

FIGURE S12: CX3CL1 led to reduced synaptic engulfment by iMGs. (A) Breakdown of **Figure 4C** highlighting the quantification of synaptoneurosomes engulfment in vehicle-treated iMGs incubated with each A_{CM} in the absence of AZD8797 treatment. **(B)** Experimental design of iMGs synaptoneurosomes phagocytosis assay upon exposure to recombinant CX3CL1 (2 ng/mL). *Schematic picture was drawn on Biorender.* **(C)** iMGs (bright field) engulfing CM-DiI-labeled synaptoneurosomes (red) during incubation with rCX3CL1. Panel depicting 0 h, 6 h, 12 h, 18 h and 24 h time-points. *Scale bar = 100 μm.* **(D)** Quantification of synaptoneurosomes phagocytosis by iMGs incubated with rCX3CL1 depicted in **(C).** Vehicle (BSA 0.1%; open circles); rCX3CL1 (2 ng/μL; filled circles). *n = 557-812 (number of cells in 4 different fields of two independent experiments).* **(E)** Breakdown of **Figure 4C** highlighting the quantification of synaptoneurosomes engulfment in AZD8797-treated iMGs incubated with each A_{CM}. *Data were analyzed by Multilevel Mixed-effects linear regression, followed by Holm-Sidak's multiple comparison test. Symbols represent Mean ± SEM. * $p < 0.05$, *** $p < 0.001$.*
